# Characterization of a novel *KCNJ2* sequence variant detected in Andersen-Tawil syndrome patients

**DOI:** 10.1186/s12881-017-0472-x

**Published:** 2017-10-10

**Authors:** Stefanie Scheiper, Brigitte Hertel, Britt-Maria Beckmann, Stefan Kääb, Gerhard Thiel, Silke Kauferstein

**Affiliations:** 1 Institute of Legal Medicine, University Hospital Frankfurt, Goethe University, Kennedyallee 104, D-60596 Frankfurt, Germany; 2 Plant Membrane Biophysics, Technical University Darmstadt, Darmstadt, Germany; 3 Department of Medicine I, University Hospital Munich, Ludwig Maximilians University, Munich, Germany

**Keywords:** Andersen-Tawil syndrome, *KCNJ2* mutation, Potassium channel Kir2.1, Functional characterization

## Abstract

**Background:**

Mutations in the *KCNJ2* gene encoding the ion channel Kir2.1 have been linked to the Andersen-Tawil syndrome (ATS). Molecular genetic screening performed in a family exhibiting clinical ATS phenotypes unmasked a novel sequence variant (c.434A > G, p.Y145C) in this gene. The aim of this study was to investigate the effect of this variant on Kir2.1 ion channel functionality.

**Methods:**

Mutant as well as wild type GFP tagged Kir2.1 channels were expressed in HEK293 cells. In order to examine the effect of the new variant, electrophysiological measurements were performed using patch clamp technique. Cellular localization of the mutant in comparison to the wild type ion channel was analyzed by confocal laser scanning microscopy.

**Results:**

The currents of cells expressing only mutant channels or a mixture of wild type and mutant were significantly reduced compared to those expressing wild type (WT) channels (*p* < 0.01). Whereas WT expressing cells exhibited at −120 mV an averaged current of −4.5 ± 1.9 nA, the mutant generates only a current of −0.17 ± 0.07 nA. A co-expression of mutant and WT channel generates only a partial rescue of the WT current. Confocal laser scanning microscopy indicated that the novel variant is not interfering with synthesis and/or protein trafficking.

**Conclusions:**

The detected sequence variant causes loss-of-function of the Kir2.1 channel and explains the clinical phenotypes observed in Andersen-Tawil syndrome patients.

## Background

The *KCNJ2* gene encodes the inwardly rectifying potassium channel Kir2.1 and is predominantly expressed in excitable tissue of heart, brain and skeletal muscle [1]. In cardiac myocytes, Kir2.1 contributes to the inwardly rectifying K^+^ current (I_K1_), which is essential for stabilizing the resting membrane potential and inducing the final repolarization phase of the cardiac action potential [2]. Mutations in the *KCNJ2* gene have been associated with Andersen-Tawil syndrome (ATS), Short QT syndrome as well as with Catecholaminergic polymorphic ventricular tachycardia (CPVT) [3–5].

ATS represents a rare hereditary disorder characterized by a triad of symptoms including cardiac arrhythmias, periodic paralysis and dysmorphic features [6]. However, most patients suffering from the syndrome do not manifest all three phenotypic features mentioned. The clinical phenotype and also the severity of the various symptoms may differ even in members of the same family [7].

Referring to the cardiac symptoms, ATS patients suffer from ventricular arrhythmias and may exhibit a prolonged QT interval in the electrocardiogram (ECG). Therefore, ATS was also classified as long QT syndrome and designated as subtype LQT7 [1]. However, in most cases, the QT prolongation is marginal. Prominent U-waves in the ECG represent an additional symptom observed in affected patients [8]. Arrhythmias may induce syncope and may eventually lead to cardiac arrest [9]. On average, cardiac abnormalities are diagnosed at an age of 13 years and need careful cardiologic monitoring [9, 10].

Periodic paralysis and muscle weakness are another manifestation of ATS. These symptoms are often observed during early childhood and their occurrence is highly variable. Paralysis attacks can last up to several days [9]. Dysmorphologies associated with the disorder include craniofacial features such as deep-set eyes, broad forehead and nose, thin upper lip, malar, maxillary and mandibular hypoplasia, dental anomalies as well as mild facial asymmetries. Additionally, small hands and feet, clinodactyly of the toes, mild syndactyly of toes two and three, and clinodactyly of the hands are common skeletal abnormalities observed in ATS patients [10].

The prevalence of ATS was estimated to be in the order of 1/1,000,000 [11]. The syndrome is inherited in an autosomal dominant fashion, but it also occurs sporadically [12]. About 60–70% of the patients exhibiting clinical ATS symptoms show genetic abnormalities in the *KCNJ2* gene, mostly resulting in complete loss-of-function [1, 13].

Molecular genetic analysis performed in a family exhibiting clinical ATS phenotypes unmasked a sequence variant in the *KCNJ2* gene (c.434A > G, p.Y145C). In the present study, we investigated the functional effect of this variant on the potassium channel Kir2.1.

## Methods

### Ethics statement

The current study was approved by the ethical commission of the University Hospital, JWG University Frankfurt (protocol number E84/06).

### Genetic analysis

Blood samples collected from individuals of the family were utilized for genetic analysis. Targeted mutational analysis of the ion channel gene *KCNJ2* (NG_008798.1) was performed using polymerase chain reaction (PCR) and standard direct sequencing, starting with the index patient. The in silico prediction tools PolyPhen-2 [14] and MutationTaster [15] were used to assess the effect of the detected sequence variant.

### Mutagenesis

For site-directed mutagenesis, the pEGFP-N2 vector comprising the wild type cDNA sequence of *KCNJ2* was used. The required sequence variant was induced by applying mutagenic primers in the thermal cycling reaction using the QuikChange II XL Site-Directed Mutagenesis Kit (Stratagene, Agilent Technologies, Waldbronn). The resulting construct was verified by sequencing.

### Electrophysiological measurements in HEK293 cells

For the functional characterization of the variant p.Y145C, the mutant as well as wild type Kir2.1 channels were expressed in human embryonic kidney (HEK) cells, cell line 293. WT and mutant were also co-transfected in a 1/1 or 1/3 ratio (wt/mut). HEK293 cells were cultivated in an incubator at 37 °C, 4–5% CO_2_ and passaged by transferring them to 35 mm plates. The cells were transfected using transfection reagent TurboFect (Thermo Fisher Scientific, Waltham, USA) and 1 μg of Kir2.1 constructs in the EGFP-N2 vector. On the next day, the cells were detached by means of accutase (PAA, GE Health Freiburg, Germany). Depending on the density of cells, 0.2, 0.3 or 0.4 ml were transferred to a new plate containing 2 ml medium.

Electrophysiological measurements were performed on single cells 2 days following transfection using conventional patch clamp technique in whole-cell configuration. Current measurements and data acquisition were carried out with an EPC-9 Patch Clamp amplifier (HEKA, Lambrecht, Germany) and the Patch Master (HEKA) software. Currents were measured at room temperature in a bath solution composed of 135 mM NaCl, 4.8 mM KCl, 1.8 mM CaCl_2,_ 1.8 mM MgCl_2_, 10 mM Glucose and 5 mM HEPES (pH 7.5). The osmolarity was adjusted to approximately 300 mosmol/kg. The pipette solution consisted of 130 mM potassium gluconate, 1 mM MgCl_2,_ 5 mM HEPES and 2 mM ATP which was adjusted to pH 7.4 and 250 mosmol/kg. To monitor Kir2.1 activity, the cells were clamped from a holding potential of −0 mV to test voltages ranging from +60 mV to −140 mV in 20 mV steps. For data analysis we used Igor Pro 6.03 software (WaveMetrics, Lake Oswego, OR).

### Data analysis

Results are reported as mean ± SD of n experiments. For statistical evaluation, the Kolmogorov–Smirnov test (KS test) was carried out to determine whether the measured data show normal distribution. Accordingly, the student’s *t*-test (one-tailed) was applied to determine the statistical significance of the results obtained.

### Confocal laser scanning microscopy

HEK293 cells were cultivated on coverslips for 2 days at 37 °C, 5% CO_2_ and transfected with GeneJuice (Novagen, Merck Chemicals Ltd., Hoddesdon, UK). Approximately 24 h following transfection, the fluorescence signal originating from the cells expressing the Kir2.1 channel tagged on the c-terminus with the enhanced green fluorescent protein (EGFP) was imaged using a Leica TCS SP5 II Confocal Systems microscope. EGFP was excited with an argon laser at 488 nm and emission recorded at 503–523 nm. Images were taken using a HCX PL APO CS 100 × 1,44 OIL UV lens and recorded under control of the Leica Confocal Software 2.00 (Leica Microsystems GmbH, Heidelberg).

## Results

### Clinical background

The index patient of the family affected by ATS (male, III.4) exhibited clinical cardiac as well as muscular symptoms at the age of 11 (Table 1). This patient and its family members underwent a complete cardiologic examination including 12-lead ECG recordings and exercise stress testing. Skeletal dysmorphic features were documented. During initial medical examination, the index patient exhibited a serum potassium concentration of 3.0 mmol/l (normal range: 3.5–5 mmol/l). Oral potassium substitution improved symptoms within hours. Afterwards, he and his mother got along with an appropriate diet as well as potassium supplements. During annually follow-up checkups, their values were in the normal range. Meanwhile the mother of the index patient (II.2) received an implantable loop recorder, because she suffered from repeated syncope during her work as a room attendant. No cardiac arrhythmias were detected during syncope. As the prominent feature in the index patient and his mother are episodes of periodic paralysis and the dysmorphic features, we classified their diagnosis as Andersen-Tawil syndrome. Since the phenotype was quite distinct, selective molecular genetic screening of the potassium channel Kir2.1 encoding gene *KCNJ2* was performed.

**Table 1:** Clinical phenotypes of the family members carrying the heterozygous mutation p.Y145C in the *KCNJ2* gene

Family member	Carrier	Year of birth	Cardiac symptoms	Age of onset	Muscular symptoms	Age of onset	Dysmorphic features
II. 2	Yes	1958	Syncope, Resting ECG with U-waves, VPB during exercise stress test starting at a heart rate of 130 bpm	14	Mild periodic paralysis of proximal upper limbs	55	Small chin
III. 2	No	1982	none	–	none	–	none
III. 3	Yes	1984	Resting ECG with U-waves	n/a	–	n/a	Small chin
III. 4	Yes	1990	Resting ECG with U-waves, VPB during exercise stress test starting at a heart rate of 120 bpm	11	Sporadic paralysis, initially only legs and later also arms affected (every few weeks to months), immediate improvement following K- and Mg- substitution	11	Very small chin, low-set ears, mild syndactyly
III. 5	Yes	1993	Resting ECG with U-waves	n/a	–	n/a	Small chin, hypertelorism, mild syndactyly

*bpm* beats per minute, *VPB* ventricular premature beats, *n/a* not applicable

### Mutation analysis

**Fig. 1:**
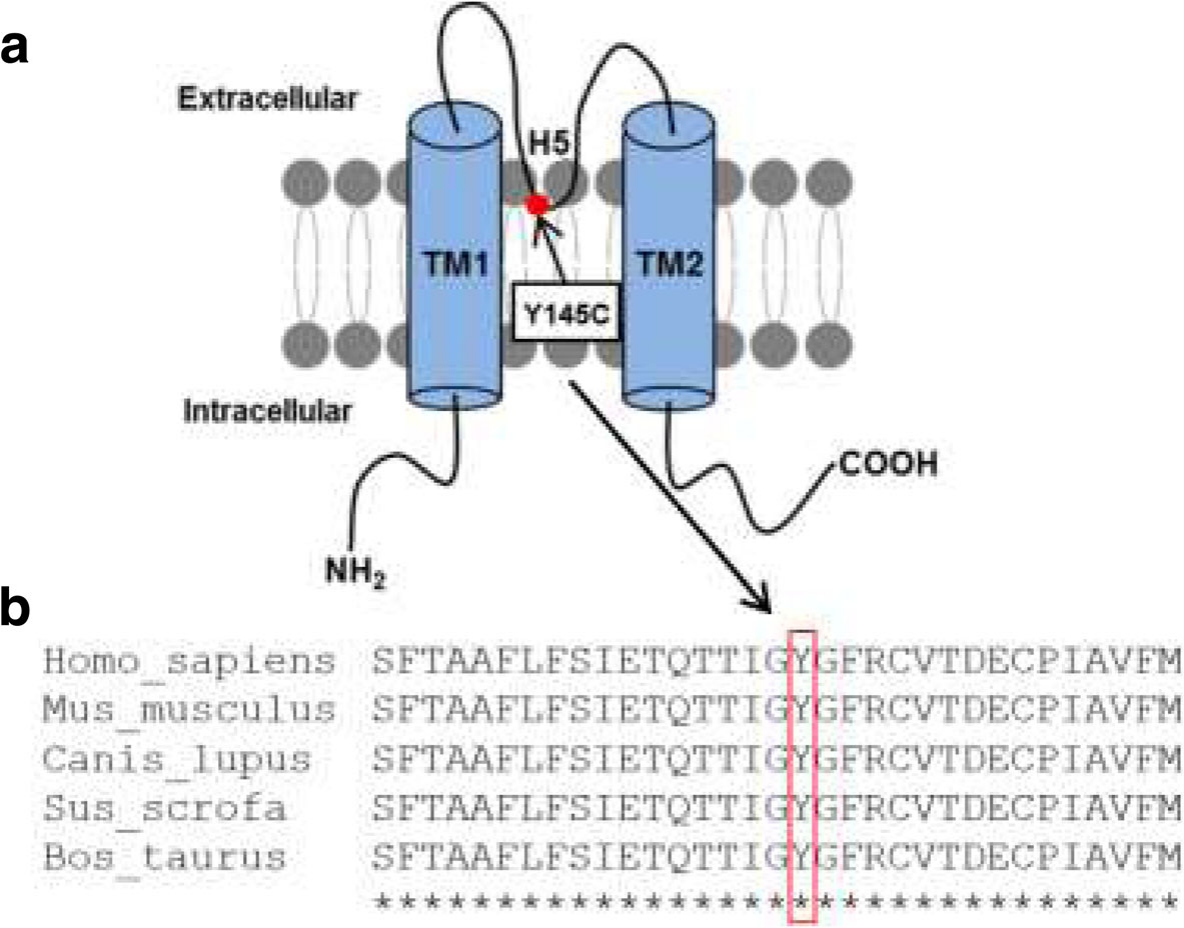
Amino acid position Y145 of the Kir2.1 channel is highly conserved in mammals. **a** Schematic representation of the localization of variant Y145C in a Kir2.1 channel subunit. The detected mutation (indicated by the red dot) results in an amino acid substitution in the pore forming region (H5). **b** Alignment of the Kir2.1 channel amino acid sequence among mammals. The alignment displays a partial sequence in human (*Homo sapiens*), mouse (*Mus musculus*), dog (*Canis lupus familiaris*), pig (*Sus scrofa*) and cattle (*Bos taurus*)

In the family affected by ATS, molecular genetic screening revealed the heterozygous point mutation c.434A > G in exon 2 of the *KCNJ2* gene leading to an amino acid substitution of tyrosine to cysteine at position 145 (p.Y145C) in the expressed ion channel protein Kir2.1. This region is located in the selectivity filter of the channel pore (Fig. 1). The ‘GYG’ signature sequence, which is highly conserved among all known potassium channels, is changed into ‘GCG’. To our knowledge, this variant is not registered in common databases (Exome Aggregation Consortium, genome Aggregation Database, NHLBI Exome Sequencing Project, NCBI dbSNP, Human Gene Mutation Database) and has not been functionally characterized so far. The novel sequence variant was expected to be pathogenic according to bioinformatics prediction tools [14, 15].

**Fig. 2:**
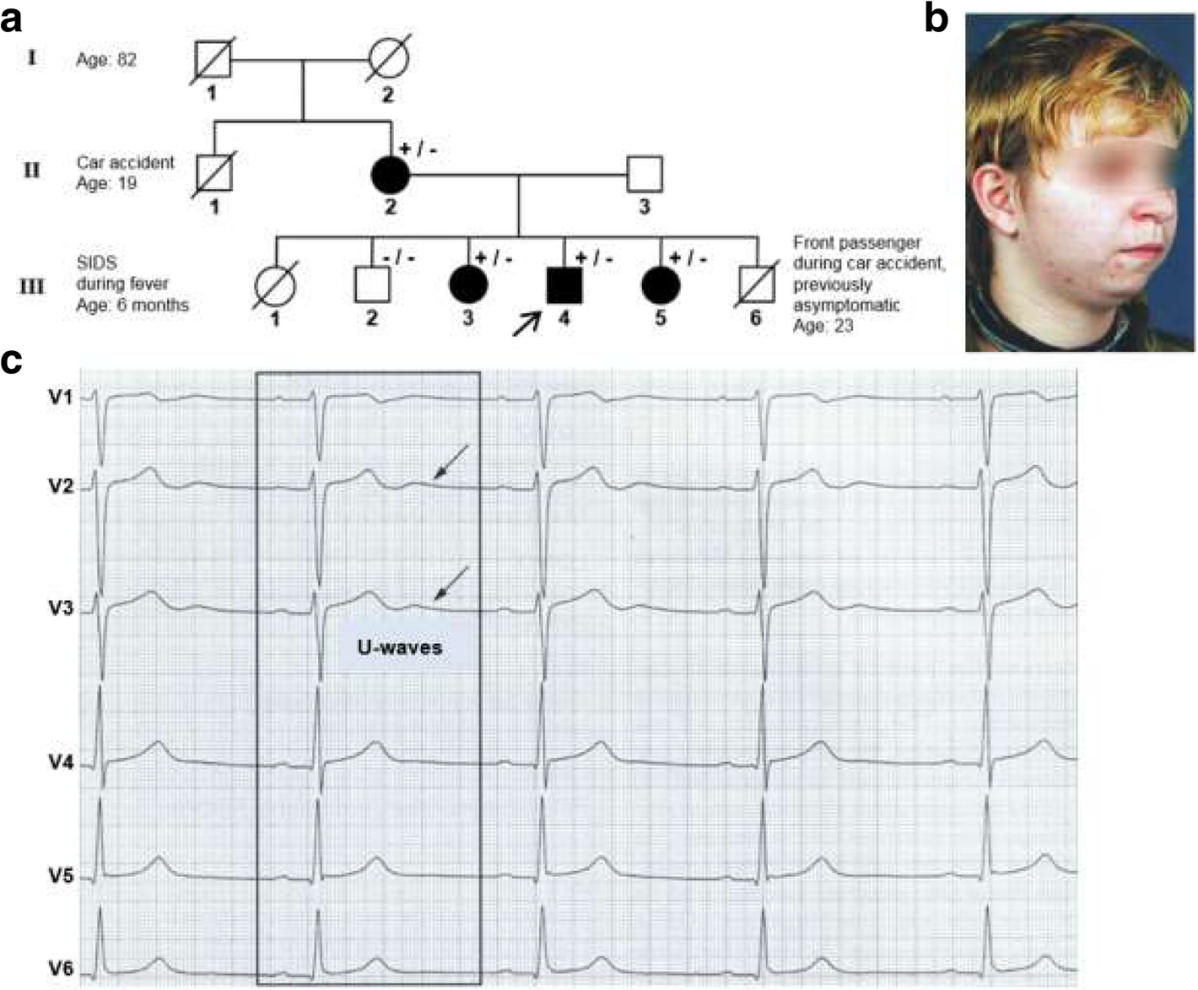
The mutant p.Y145C was detected in a family with diagnosed ATS. **a** Pedigree of the family. Filled symbols indicate clinically affected individuals. +/− represents heterozygous carriers of mutation Y145C in KCNJ2 and −/− indicates exclusion of Y145C. Crossed symbols represent deceased family members. **b** ATS index patient (arrow in **a**) exhibiting typical clinical symptoms such as small chin, low-set ears and hypertelorism (not shown) **c** Prominent U-waves in resting ECG (scale 50 mm/s) of the ATS patient. Reprinted with permission from Beckmann and Kääb as well as the Hans Marseille Verlag [21]

All family members carrying this sequence variant exhibit characteristic phenotypes of this rare hereditary disease. They present clinical symptoms such as U-waves in the resting ECG, ventricular premature beats during exercise stress test, periodic paralyses as well as dysmorphic features including small chin, low-set ears, hypertelorism and syndactyly (Table 1, Fig. 2).

### Effect of the novel sequence variant on Kir2.1 channel conductance

In order to examine the effect of variant p.Y145C on the Kir2.1 channel conductance, electrophysiological measurements were performed. Therefore, HEK293 cells were transfected using the pEGFP-N2 vector comprising either the *KCNJ2* wild type cDNA sequence or the mutated sequence (c.434A > G), or were co-transfected with both constructs. By measuring the whole cell currents of cells, which express either the WT channel or its mutant or a mixture of both, we obtain information on the functional impact of the novel sequence variant.

**Fig. 3:**
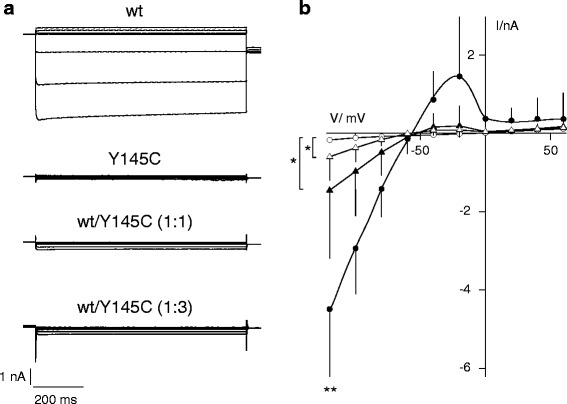
Representative currents and current/voltage relations recorded from HEK293 cells expressing different Kir2.1 constructs. **a** Current traces of HEK293 cells expressing WT Kir2.1 channels or the Y145C mutant alone. WT and mutant were also co-transfected in a 1/1 or 1/3 ratio (wt/mut). **b** Mean current/voltage relations (± standard deviation) from measurements as in **a** from HEK293 cells expressing WT (●), mutant (○) or a 1:1 (▲) or 1:3 mixture (△) of WT and mutant channels. The mean value of the WT current at −120 mV is significantly higher (*p* < 0.01, **) than any of the other constructs. At −120 mV, co-expression of WT and mutant generates an inward current, which is significantly higher (*p* < 0.05; *) than those measured with the mutant channel only

Cells expressing the wild type Kir2.1 channel showed the expected features of a Kir type inward rectifier. Clamp voltages negative of approximately −20 mV elicit currents, which are first outward. They reverse at the K^+^ equilibrium voltage to produce large inward currents with increasing negative clamp voltages. Representative current traces as well as the mean steady state I/V relation from 5 cells are shown in Fig. 3. In contrast to cells expressing the WT channel, mutant transfected cells exhibit almost no current. Even at negative potentials, the currents in these cells are minute and reveal no apparent inward rectification (Fig. 3).

To simulate the allelic heterozygosity, HEK293 cells were co-transfected with WT and Y145C mutant channel (wt/mut) at a 1/1 or 1/3 ratio with a total of 1 μg DNA per dish. Representative currents and the mean current/voltage relations from measurements in co-transfected cells are shown in Fig. 3 a, b. The latter currents and I/V relations are different from those recorded in cells with either WT channel or mutant channel only. The currents at −120 mV generated by co-expression (1/1: −1.5 ± 1.8 nA (*n* = 11); 1/3: −0.6 ± 0.6 nA (*n* = 9)) are significantly smaller than currents produced by the WT channel (−4.5 ± 1.9 nA (*n* = 5)). Unlike the currents measured in cells expressing the mutant channel only (−0.17 ± 0.07 nA (*n* = 8)) a co-expression of WT and mutant causes a small but Kir2.1 typical inward rectifying current. The results of these experiments imply that the pore mutation has a dominant negative effect on the Kir2.1 function. A co-expression with WT channels, which mimics allelic heterozygosity, generates a small Kir2.1 current meaning that a co-expression is not able to rescue WT channel activity. At moderate membrane voltage, e.g. voltages which are relevant for the free running voltage, the Kir2.1 mediated outward current is about 5-fold lower (Fig. 3b) than that produced by the WT channel.

### Cellular localization of Kir2.1 WT and p.Y145C mutant channels

**Fig. 4:**
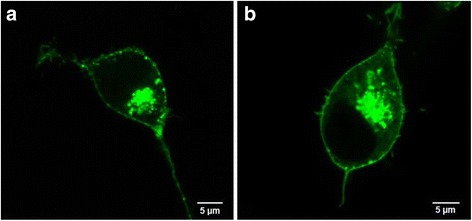
Cellular localization of GFP tagged wild type and mutant Kir2.1 channels expressed in HEK293 cells. **a** EGFP fluorescence signal of a cell expressing Kir2.1 wild type. **b** EGFP fluorescence signal of a cell expressing Kir2.1 mutant p.Y145C. Both images were taken in the equatorial plane of cells using confocal laser scanning microscopy

The electrophysiological measurements indicated that the expression of mutant Kir2.1 channels in HEK293 cells results in impaired conductivity. In order to investigate, whether the sequence variant interferes with protein trafficking, confocal laser scanning microscopy was performed. HEK293 cells were transfected with either WT or mutant GFP tagged Kir2.1 channels and analyzed for the cellular localization of the channel protein. Figure 4 shows the fluorescence signal of EGFP in exemplary cells, which express either the WT or the mutant channel. The cellular distribution of the fluorescence is indistinguishable between both cells. Both exhibit a central fluorescent structure, which presumably reflects the Golgi apparatus; the latter is generally visible in cells, which overexpress a protein. More important for the present data is that the green fluorescence is localized at the cell membrane in both cases; the membrane localization is well visible in the filopodia of the cells, which are mainly formed by plasma membrane. The results indicate that the WT and the mutant channel show no appreciable difference in trafficking of the channel protein to the plasma membrane on this level of resolution.

## Discussion

Mutations in the *KCNJ2* gene have been reported to cause Andersen-Tawil syndrome [3, 4, 16]. About 60–70% of the patients exhibiting clinical ATS symptoms show abnormalities in this gene [1, 13].

Here we report a novel *KCNJ2* sequence variant (p.Y145C) in a family with diagnosed ATS. All family members screened and presenting clinical phenotypes of this rare hereditary disease are heterozygous carriers of the new variant. The fact that the family member, who is not carrying the missense variant, is not exhibiting any ATS symptoms suggests that the new variant localized in the channel pore of the Kir2.1 channel contributes to the symptoms in ATS patients.

All registered mutations localized in the pore forming region of the Kir2.1 channel and adjacent to variant p.Y145C have been classified as pathogenic. The p.Y145C variant is localized in the signature sequence of K^+^ channels, which forms the selectivity filter of these channels and which is essential for proper channel function. Mutations in this domain, namely at amino acid positions 144 and 146 in the Kir2.1 channel, have already been declared as ‘hotspots’ referring to the Andersen-Tawil syndrome [9]. Based on these facts, it was already assumed that the new sequence variant, which we report here, corrupts ion channel function and constitutes to the Andersen-Tawil phenotypes of the patients carrying this sequence aberration.

Functional analysis of cells expressing WT and mutant Kir2.1 channel confirms that the mutant Kir2.1 channel is indeed functionally impaired. In contrast to the large inward currents detected in cells expressing the wild type channel, the mutant channels generated no appreciable inwardly rectifying current. Also in co-expression experiments, the pore mutation showed a dominant negative effect on ion channel function. The current response of cells expressing only the mutant channel was just slightly higher than the current response observed in non-transfected cells. This conductance is not voltage dependent and hence not caused by Kir2.1 activity. It is more likely an unspecific side effect of an overexpression of channel protein. Up to now, all *KCNJ2* mutations, which were functionally characterized and associated with the Andersen-Tawil syndrome, resulted in complete loss-of-function when expressed as homotetramers [16]. The amino acid substitution of tyrosine by cysteine in the selectivity filter corrupts the delicate structure for ion permeation rendering the channel nonfunctional. The same non-functional Kir2.1 channels were already reported as a consequence of mutations in close vicinity to the p.Y145C variant [9]. Co-expression experiments of adjacent variants also showed a dominant negative impact on channel function [1]. Dart et al. [17] obtained similar results when they expressed the mutant Y145C in the murine potassium channel Kir2.1 in Chinese hamster ovary (CHO) cells to determine the characteristics of the pore lining residues.

Aberrant ion channel functionality is not always based on conduction defects. Bendahhou et al. [18] showed that sequence aberrations in Kir2.1 channels may also result in impaired channel trafficking to the cell surface. Taking this into account, the patch-clamp experiments can be interpreted in two ways, namely, in that the mutation renders the channel inactive or that it impairs synthesis and/or trafficking of the protein to the plasma membrane. Given the fact that the mutation is localized right in the most conserved domain of K^+^ channels and that nearly all mutations in this domain impair channel function [19], it is most likely to assume that the Y145C mutant is inactive. To examine this hypothesis, we subsequently tested whether the sequence variant interferes with protein trafficking by confocal laser scanning microscopy using GFP tagged channel proteins. The analysis of the confocal images shows no obvious differences between the WT and mutant channel proteins. Both exhibit a similar fluorescence at the plasma membrane indicating that the new variant Y145C is not interfering with protein trafficking. This again supports the assumption that the new variant impairs Kir2.1 channel conductance and hence reduced inwardly rectifying (I_K1_) currents.

Referring to the clinical ATS phenotypes in the affected family, all Y145C carriers exhibited a resting ECG with prominent U-waves. This can be explained by a reduction of I_K1_ currents due to mutant Kir2.1 channel expression [9]. Defects in Kir2.1 channels have also been shown to trigger an increased frequency of spontaneous action potentials [10]. This could explain the occurrence of ventricular premature beats in case of two ATS patients. Moreover, a reduced I_K1_ current in skeletal muscle tissues may result in an imbalance of potassium ions and reduced membrane excitability leading to muscle weakness and periodic paralysis [9]. These symptoms have been linked to ATS and were also observed in the ATS patients carrying the Kir2.1 channel variant Y145C. In our patients oral potassium supplementation resulted in resolution of paralysis within hours. As the patients do not suffer from serious cardiac symptoms showing few ventricular premature beats only until now, the current risk to develop a tachycardiomyopathy or a life threatening arrhythmia is considered to be low and cardiological examinations including echocardiographic screening is performed on a regular basis with reassessment of the risk. Therefore, no specific therapy has been initiated next to potassium supplementation until now. In the case of patient II.2, which represents the mother of the index patient, an implantable loop recorder has been implanted meanwhile due to the occurrence of presyncopes and syncopes and again she suffered from repeated syncope. However, during syncope no cardiac arrhythmias were documented. Recently, Tully et al. [20] described the detection of the novel variant in a family with diagnosed ATS and concluded that the disease is likely caused by the variation. Our results verify the authors’ assumption that this variant results in clinical ATS phenotypes.

## Conclusion

A novel sequence variant (p.Y145C) localized in the highly conserved pore forming region of the potassium channel Kir2.1 was identified. This variant was detected in a family exhibiting phenotypes of Andersen-Tawil syndrome. Functional characterization of this variant indicated that the mutation results in loss-of-function of the Kir2.1 channel. The channel mutant generated no appreciable current when expressed as homotetramer in HEK293 cells. This lack of conductance must be caused by a corruption of channel function as a result of the mutation in the selectivity filter. A co-expression of the WT channel with the mutant, a condition, which simulates the heterozygous condition, causes a strong reduction of the channel current suggesting a strong dominant negative effect of the pore mutation on Kir2.1 function in a channel tetramer. Trafficking of the mutant to the plasma membrane appears largely unaffected; confocal images of GFP tagged channel proteins revealed no differences between WT and mutant Kir2.1 proteins. The results suggest that mutant Y145C in the Kir2.1 channel can be associated with the clinical phenotypes of the Andersen-Tawil syndrome patients. These findings may help to improve future genetic counselling and medical treatment of patients carrying this variant.

## Abbreviations

ATS: Andersen-Tawil syndrome
bpm: beats per minute
CLSM: Confocal laser scanning microscopy
ECG: Electrocardiogram
EGFP: Enhanced green fluorescent protein
HEK293: Human embryonic kidney cells (cell line 293)
VPB: Ventricular premature beats
WT: Wild type
